# A Multi-Targeting Approach to Fight SARS-CoV-2 Attachment

**DOI:** 10.3389/fmolb.2020.00186

**Published:** 2020-08-03

**Authors:** Luciano Pirone, Annarita Del Gatto, Sonia Di Gaetano, Michele Saviano, Domenica Capasso, Laura Zaccaro, Emilia Pedone

**Affiliations:** ^1^Institute of Biostructures and Bioimaging, CNR, Naples, Italy; ^2^CIRPEB, University of Naples Federico II, Naples, Italy; ^3^Institute of Crystallography, CNR, Bari, Italy; ^4^CESTEV, University of Naples Federico II, Naples, Italy

**Keywords:** spike protein, galectin, integrin, ACE2, ganglioside

## Abstract

The public health has declared an international state of emergency due to the spread of a new coronavirus (SARS-CoV-2) representing a real pandemic threat so that to find potential therapeutic agents is a dire need. To this aim, the SARS-CoV-2 spike (S) glycoprotein represents a crucial target for vaccines, therapeutic antibodies, and diagnostics. Since virus binding to ACE-2 alone could not be sufficient to justify such severe infection, in order to facilitate medical countermeasure development and to search for new targets, two further regions of S protein have been taken into consideration here. One is represented by the recently identified ganglioside binding site, exactly localized in our study in the galectin-like domain, and the other one by the putative integrin binding sites contained in the RBD. We propose that a cooperating therapy using inhibitors against multiple targets altogether i.e., ACE2, integrins and sugars could be definitely more effective.

## Introduction

The novel coronavirus SARS-CoV-2, whose infection is associated to a respiratory syndrome with impaired lung and alveolar function, can lead to acute respiratory failure and even death (Kanduca and Shoenfeldb, 2020; Wrapp et al., 2020) so to be declared as a Public Health Emergency of International Concern by the World Health Organization.

The novel virus belongs to the betacoronavirus genus, but differently from other less pathogenic human CoVs such as HCoV-OC43 and HCoV-229E, it has a strong pathogenicity that leads to death.

So far the molecular bases and the mechanism that link SARS-CoV-2 coronavirus infection to the pulmonary pathology are still unexplored (Tai et al., 2020).

HCoVs are positive-sense, single-stranded RNA viruses and 30,000 bp long. Two types of proteins are identified in HCoVs: four structural proteins including spike (S), envelope (E), membrane (M), and nucleocapsid (N) proteins and non-structural proteins, like proteases (nsp3 and nsp5) and RNA dependent-RNA polymerase (nsp12). The S protein is a pivotal tool for virus adhesion and entry into host cells and it can represent an intriguing target for the development of antibodies, entry inhibitors and vaccines. It is present on the virion’s outer surface and displays a homo-trimeric state.

Protein S allows viral entrance into host cells by firstly binding to a host receptor, such interaction occurring through the receptor binding domain (RBD) in subunit S1, and secondly through subunit S2 the viral and host membranes fuse. In particular it is known that similar to SARS-CoV, SARS-CoV-2 also recognizes angiotensin-converting enzyme 2 (ACE2) as its host receptor binding (Ibrahim et al., 2020; Tai et al., 2020; Walls et al., 2020).

The emerging worldwide spread of SARS-CoV-2 and its hard impact on public health urgently require an effort to increase understanding of the entry mechanism of the virus into host cells, so to explain the advanced transmission rate from person to person.

Coronaviruses could have acquired different approaches for starting the infection. This is true for both the attachment and the following membrane fusion step and the only binding to ACE-2 might not be enough to justify such severe infection of respiratory airways. Thus, it is likely that SARS-CoV-2 might also bind to other cell surface attachment factors (Peng et al., 2012; Fantini et al., 2020).

In this context, we focused on further emerging novel spike protein targets, such as receptors belonging to the family of integrins or sialic-acid-containing cell surface structures, considering that sialic acids linked to glycoproteins and gangliosides as well as integrins are employed by a wide spectrum of viruses, including coronaviruses, as receptors and/or attachment factors for cell entry (Wei et al., 2014; Matrosovich et al., 2015; Teoh et al., 2015).

### A New Putative Target: The Galectin-Like Domain

The receptor-binding S1 subunit of S protein is constituted by two independent domains, the N-terminal domain (S1-NTD) and the C-terminal domain (S1-CTD). One or both of these S1 domains potentially bind receptors and function as RBD (Li, 2016).

S1-NTDs, which unexpectedly contain the same fold as human galectins (galactose-binding lectins), are responsible for binding sugar and function as viral lectins (Peng et al., 2012).

The crystal structure of the spike protein NTD of bovine coronavirus (BCoV NTD) has been solved (Peng et al., 2012). It consists of a beta-sandwich core structure containing one six-stranded beta-sheet and one seven-stranded beta-sheet that are held together by hydrophobic interactions. This core structure has the same fold as human galectins (Peng et al., 2012). The sugar-binding site in BCoV NTD has been identified by alanine scanning of residues potentially involved. In particular, critical sugar-binding residues resulted to be Y162, E182, W184, and H185, spread on two loops, suggesting that the pocket above the beta-sandwich core is the sugar-binding site. Furthermore, a glycan screen arrays allowed to identify, among 611 types of sugar, the 5-N-acetyl-9-O-acetylneuraminic acid (Neu5,9Ac2) as favorite sugar for BCoV NTD. Although BCoV NTD and human galectins bind different carbohydrates, they present a similar binding domain.

The HCoV-OC43 NTD structure has not yet been obtained, but contains the same galectin-like domain (Peng et al., 2012) and, considering its highly sequence homology with BCoV NTD structure, it is plausible to imagine that it could bind the same sugar (Neu5,9Ac2).

An important feature of SARS-CoV-2, being a betacoronavirus, is that, in addition to the protein membrane receptor, its attachment also is dependent on glycoproteins and gangliosides containing sialic acid which act as main attack sites along the respiratory tract (Matrosovich et al., 2015).

Interestingly, a recent paper by Fantini et al. (2020) reported a study where a new type of ganglioside-binding domain (GBD) in the NTD of the SARS-CoV-2 S protein was identified. In particular, this domain resulted to be spanning from aa 111 to aa 158, and entirely conserved among clinical isolates globally, thus possibly improving the attachment of the virus to lipid rafts and facilitating the interaction with the ACE-2 receptor. In addition, the authors demonstrated, by means of structural and molecular modeling techniques, that in the presence of chloroquine (CLQ), the S protein does not retain the ability to bind gangliosides, so justifying the action of CLQ, one drug used for COVID-19 therapy.

It is worth noting that the GBD is fully conserved also in the NTD of the Bat RaTG13, further demonstrating a close relationship between the bat and the human coronavirus currently isolated in the world. However, this domain being a bit different in other bat and human CoVs, it suggests a recent evolution which could clarify the reason why SARS-CoV-2 results more infectious.

**FIGURE 1:**
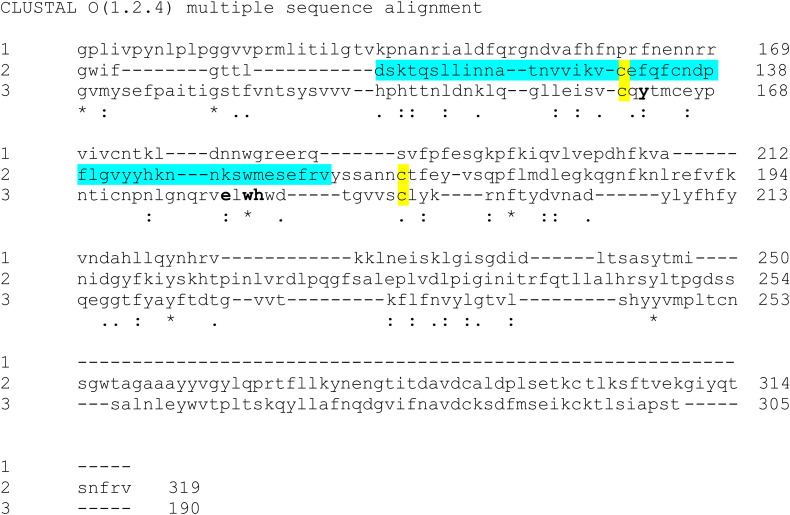
Sequences alignment by Clustal omega (https://www.ebi.ac.uk/Tools/msa/clustalo/). (1) CRD from human Galectin 3 – BAA22164 Genebank; (2) SARS CoV-2 NTD_103aa–314aa_ PDB:6VSB; (3) BCoV NTD_116aa–305aa_ PDB: 4H14. The ganglioside domain in SARS CoV-2 NTD is in cyan (Fantini et al., 2020). The residues critical for sugar-binding are reported in bold (as shown in Peng et al., 2012 regarding BCoV sugar binding site). The conserved cysteine residues between SARS CoV-2 and BCoV NTD are shown in yellow.

What is extremely interesting is to observe, for the first time, that the ganglioside domain seems to reside exactly in the galectin-like domain as deduced comparing the sequences of the Carbohydrate Recognition Domain (CRD) of the human galectin 3 (Di Gaetano et al., 2019) with the corresponding regions in the NTDs from SARS-CoV-2 and bovine coronavirus (Figure 1). In addition it can be stated that not only the beta-sandwich core of the sugar-binding site is conserved, but also the structural constraint represented by the disulphide bond between C130/C165 in the NTD from SARS-CoV-2 and the corresponding residues C160/C193 in the BCoV galectin-like domain. Moreover, even more intriguing, we can claim that all the peculiar residues for binding the sugar are also preserved i.e., Y162, E182, W184 and H185 in BCoV sugar binding site (Peng et al., 2012) as highlighted in Figure 1. Another evidence, strengthening our hypothesis that the GBD is localized in the galectin-like fold, is that this domain from SARS-CoV-2 is able to bind N-Acetylneuraminic acid (Neu5Ac), the main sialic acid present in human glycoproteins and gangliosides, exactly like the galectin-like domain from BCoV NTD is able to bind Neu5,9Ac2 (Peng et al., 2012).

**FIGURE 2:**
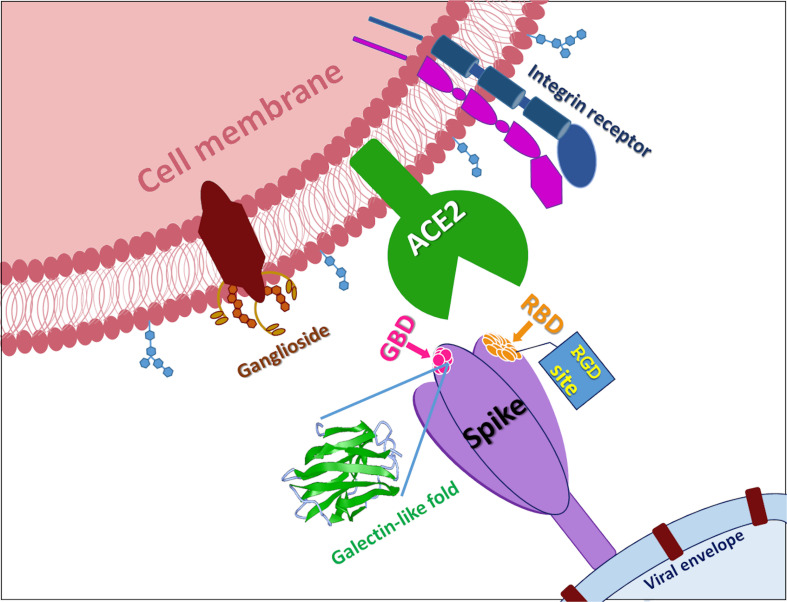
SARS-CoV-2 S-protein. In the figure is reported a cartoon that shows the multiple attachment points of SARS-CoV-2 spike protein. RBD is receptor binding domain, GBD is ganglioside binding domain, RGD site is the classic tripeptide recognized by integrins, ACE2 is Angiotensin I Converting Enzyme 2.

Therefore, altogether these data prompt us to suggest that the galectin-like domain could represent an interesting and novel target beyond the ACE2 binding domain (Figure 2). Its high structural homology with the galectin-domain and the already available structural information on this fold could help to drive a rational design of inhibitors of different nature such as glycomimetics and organic molecules aimed at blocking the interaction with any protein/sugar, a necessary step for the adhesion of the virus to human cells.

### A Further New Putative Target: Integrin Domain

Recent evidences showed that another interesting “door” for SARS-CoV-2 to enter the cell could be through integrin receptors. There are several integrin-dependent viral infection strategies in which integrins can be employed as principal adhesion receptors or as co-receptors in the entrance (Gao et al., 2008).

The variation of crucial residues in S protein of SARS-CoV-2 may contribute to the highly transmission efficiency of the virus. The reported evolutionary mutation of K403R in S1 protein of SARS-CoV-2 forms an RGD motif in RBD at the interaction surface neither been found in Bat RaTG13 nor in SARS-CoV (Yan et al., 2020; Figure 2). The RGD motif is the cell attachment site of a great amount of adhesive extracellular matrix and cell surface proteins and is recognized by the membrane receptor integrins. The integrins are heterodimers of α- and β-subunits linked in a non-covalent manner and play key functions in cell adhesion, cell-cell interactions, signaling and defense mechanisms. Human metapneumovirus is similar to SARS-CoV-2 regarding the organ tropism and symptoms, and its protein F’s RGD triade displays a fold comparable to that of SARS-CoV-2 (Chang et al., 2012; Wei et al., 2014).

Integrins play an important role in different respiratory diseases, in particular in pneumonia resulting from bacteria or virus infections (Teoh et al., 2015). Given these evidences, we can speculate that it is plausible that SARS-CoV-2 acquired integrin-binding to promote entry into host cells and, thus, integrins can be considered a novel challenging target against the virus. In addition to interacting with ACE2, S protein may also be recognized by integrins in alveolar epithelial cells through its RGD sequence to accelerate the infection process. Upon the binding, the RBD of S1 subunit undergoes a conformational shift and this change exposes or hides the key region of binding domain to access to ACE2 (Wrapp et al., 2020). In this scenario, the RGD motif would be exposed to the surface of the host cell membrane with the key binding region and thus prones to interact with integrin. Literature data show that also ACE2 displays a conserved RGD motif and it is able to bind integrin subunits. The binding seems to be RGD-independent because of the inaccessibility of the motif, letting to speculate that thanks to a conformational shift the site could be exposed, enhancing cell adhesion and therefore justifying the higher infectivity. In addition, the tripeptide LDI, homolog to LDV sequence recognized by some integrin sub-families, is also present in the spike glycoproteins of SARS-CoV-2, SARS-CoV, and Bat RaTG137 (Tresoldi et al., 2020). All these indications lead us to think that selective agents able to block integrin binding (Del Gatto et al., 2006; Farina et al., 2016; Comegna et al., 2017; Di Gaetano et al., 2018) could represent a further appealing approach to prevent virus cell attachment and to neutralize the pathogen.

## Discussion

A lot of viruses employ numerous receptors and/or co-receptors for penetrating into host cells (Jeffers et al., 2004). These let hypothesize not only that multiple interactions of a viral envelope glycoprotein with different receptors favor the infection of various tissues or hosts, but also that diverse S- receptor interactions can be described sequentially on a single cell type or possibly on different cell types.

Our idea is that these emerging new spike targets above cited can be reasonably justified by the fact that, as it is well- known, the S of coronaviruses are defined as some of the biggest viral spike glycoproteins known, so it is plausible to imagine that different domains within a single S protein could interact with various receptors rendering easier and faster the entry of the virus. This aspect is crucial because, while it is ascertained that ACE2 is the most recognized door for the entry, on the other side to target only ACE2 could be limiting considering that recent data show that ACE2 is up-regulated in diabetes and treatment with ACE inhibitors and angiotensin II type-I receptor blockers (Fang et al., 2020). Therefore, it would mean that an increased expression of ACE2 would be associated to an easier infection with SARS-CoV-2.

Accordingly, diabetes and hypertension treatment with ACE2-stimulating drugs could increase the risk of serious and fatal COVID-19 development. Therefore, this aspect suggests that a COVID-19 therapy by a multi-targeting approach is the right way and the above reported systems in our opinion could represent sound therapeutic co-receptors to target. Moreover, the demonstrated competitive action of CLQ for the ganglioside site suggests a valid base on the important role that to inhibit the interaction with ganglioside could play in the COVID-19 therapy; such indication prompts us to strongly consider that ligands modulating the interaction with integrins (Del Gatto et al., 2006; Farina et al., 2016; Comegna et al., 2017; Di Gaetano et al., 2018; Fang et al., 2020) or, at the same level, with a sugar through the galectin-domain could represent a suitable therapeutic application. It is important to underline that SARS-CoV-2 S protein-targeting monoclonal antibodies (mAbs) with potent neutralizing activity, a promising therapeutic strategy against COVID-19, target not only the RBD, but also the NTD region (Chi et al., 2020). In particular a newly identified mAb, named 4A8, interacts with residues (Y145, K147, K150, and W152) exactly located in the GBD domain (Figure 1; Chi et al., 2020). Differently, Cao et al. (2020) identified 72 non-RBD binding mAbs exhibiting no pseudovirus neutralizing ability, suggesting once more other regions used by the virus to interact with the cells. In addition a newly identified mutation, D614G (Zhang et al., 2020) localized out of the RBD region, has an impact on the increase of infectivity, highlighting also the involvement of further regions in the enhanced infection. Altogether, these indications open a new scenario for the future COVID-19 treatment where a multi-targeting therapy could be applied considering ACE2, integrin and galectin domain inhibitors.

## Data Availability Statement

The datasets presented in this study can be found in online repositories. The names of the repository/repositories and accession number(s) can be found in the article/supplementary material.

## Author Contributions

All authors listed have made a substantial, direct and intellectual contribution to the work, and approved it for publication.

## Conflict of Interest

The authors declare that the research was conducted in the absence of any commercial or financial relationships that could be construed as a potential conflict of interest.
